# Nephronectin mediates p38 MAPK‐induced cell viability via its integrin‐binding enhancer motif

**DOI:** 10.1002/2211-5463.12544

**Published:** 2018-11-15

**Authors:** Jimita Toraskar, Synnøve N. Magnussen, Konika Chawla, Gunbjørg Svineng, Tonje S. Steigedal

**Affiliations:** ^1^ Department of Clinical and Molecular Medicine Faculty of Medicine and Health Sciences Norwegian University of Science and Technology (NTNU) Trondheim Norway; ^2^ Central Norway Regional Health Authority Stjørdal Norway; ^3^ Department of Medical Biology Faculty of Health Sciences UiT‐The Arctic University of Norway Tromsø Norway; ^4^ Bioinformatics Core Facility‐BioCore Norwegian University of Science and Technology (NTNU) Trondheim Norway

**Keywords:** breast cancer, cell viability, extracellular matrix, integrin, nephronectin, p38 MAPK

## Abstract

Nephronectin (NPNT) is an extracellular matrix (ECM) protein involved in kidney development. We recently reported intracellular NPNT as a potential prognostic marker in breast cancer and that NPNT promotes metastasis in an integrin‐dependent manner. Here, we used reverse‐phase protein array (RPPA) to analyze NPNT‐triggered intracellular signaling in the 66cl4 mouse breast cancer cell line. The results showed that the integrin‐binding enhancer motif is important for the cellular effects upon NPNT interaction with its receptors, including phosphorylation of p38 mitogen‐activated protein kinase (MAPK). Furthermore, analysis using prediction tools suggests involvement of NPNT in promoting cell viability. In conclusion, our results indicate that NPNT, via its integrin‐binding motifs, promotes cell viability through phosphorylation of p38 MAPK.

**Abbreviations**
AIA: mutated EIE motif of nephronectin
ECM: extracellular matrix
EV: empty vector
IPA: ingenuity pathway analysis
MAPK: mitogen‐activated protein kinase
NPNT: nephronectin
RGE: mutated RGD motif of nephronectin
rmNPNT: recombinant nephronectin
RPPA: reverse‐phase protein array

The cell–extracellular matrix (ECM) interaction plays a vital role in tissue homeostasis, as well as in determining the fate of cancer cells 1. The composition of the ECM and competitive binding among integrins determines whether cells survive, differentiate, proliferate, migrate, or influence shape and cell polarity (reviewed in 2, 3, 4). Integrins are transmembrane receptors well known for their ability to link ECM ligands to the cytoskeleton and transduce signals, which effects cellular responses. Several integrins (α8β1, αVβ3, αVβ5, αVβ6, and α4β7) are shown to bind to NPNT 5, 6, where some are known to bind the common RGD (Arg‐Gly‐Asp) integrin‐binding motif 2. NPNT contains an additional integrin‐binding motif, known as the EIE (Glu‐Ile‐Glu) enhancer motif, located downstream of the RGD motif and known to interact mainly with integrin α8β1 7, 8. ECM–integrin interactions are known to influence breast cancer progression, and altered expression of integrins may predict poor survival in breast cancer 9, 10. Also, in breast cancer tissues the expression of ECM components is often elevated compared to normal tissues 11. High expression levels of NPNT have been linked to the metastatic propensity of mouse breast cancer cells in a model of spontaneous metastasis 12. In a different syngeneic mouse model of breast cancer, higher levels of NPNT were reported in metastatic mammary tumor cells compared to nonmetastatic cells 13. Our recent results show that NPNT overexpressing 66cl4 cells (66cl4‐NPNT) have an increased ability to form lung metastases compared to 66cl4‐empty vector cells (66cl4‐EV) in an experimental metastasis assay. A single point mutation of the RGD motif alone (66cl4‐RGE) was not sufficient to reduce the number of NPNT‐induced metastatic lesions. However, tumor burden was significantly reduced in mice injected with cells overexpressing NPNT mutated in both the RGD and enhancer EIE motif (66cl4‐RGE‐AIA). This highlights the importance of NPNT–integrin interaction in the formation of lung metastasis 14. The current study aimed to investigate the biological function of NPNT in the 66cl4 cell line. We performed a comprehensive analysis using reverse‐phase protein array (RPPA) to further identify molecular signals triggered by the NPNT–integrin interaction. Using *in vitro* assays, we confirm the involvement of NPNT in promoting cell viability.

## Materials and methods

### Cell culture

As described previously, 66cl4 cells were stably transduced to overexpress NPNT or NPNT mutants (RGE or RGE‐AIA), while 66cl4 empty vector cells (EV) were used as a control 14. Furthermore, shRNA was used to knock down NPNT protein levels in 4T1 cells (sh‐NPNT), while a nontargeting shRNA in 4T1 cells was used as a control (sh‐ctr) 14. All cell lines were cultured in (1X) minimum essential medium α (Thermo Fisher Scientific, Cat: 22561021), supplemented with 10% fetal bovine serum, 1% (v/v) penicillin–streptomycin, and 1M hepes buffer (Thermo Fisher Scientific, Cat: 15630080). Cell lines were routinely tested for mycoplasma infection.

### Immunofluorescence

66cl4‐EV and 66cl4‐NPNT were cultured for 24 h in serum‐free medium to evaluate the cell surface localization of NPNT. 66cl4‐EV cells were used as negative control. The effect of incubating 66cl4‐EV cells with 2 μg·mL^−1^ recombinant mouse NPNT (rmNPNT) (R&D systems, Minneapolis, MN, USA; Cat: 4298‐NP‐050) in PBS for 1 h prior fixing was also investigated. Cells were fixed with 4% paraformaldehyde (PFA). Permeabilization of cells was avoided to visualize extracellular NPNT. Anticollagen V (Abcam, Cambridge, MA, USA; Cat: ab7046) was used as a positive control. NPNT was identified with anti‐NPNT (Abnova, Taipei, Taiwan; Cat: PAB8467) (1 : 150) and Alexa Fluor^®^ 488 as secondary antibody (Abcam, Cat: ab150077) (1 : 1000). Images were captured using confocal laser scanning microscope (Zeiss LSM 510 Meta, Oberkochen, Germany). Hoechst was used to stain the nucleus.

### Reverse‐phase protein array

66cl4‐EV, 66cl4‐NPNT, 66cl4‐RGE, and 66cl4‐RGE‐AIA cells were grown in serum‐free medium for 24 h and then collected at 80% confluency using a cell scraper and snap‐frozen in liquid nitrogen. To investigate the effect of rmNPNT, 66cl4‐EV cells were seeded on plates precoated with 2 μg·μL^−1^ rmNPNT in serum‐free medium for 24 h. Control plates were preincubated with PBS alone. Frozen cell pellets (more than 1 million cells) were analyzed at the MD Anderson Cancer Center, RPPA core facility, USA. RPPA is high‐throughput functional proteomics analysis designed to analyze cellular protein activity in signaling networks by measuring protein levels (both total and phosphorylated forms) using high‐quality validated antibodies 15, 16. Considering values from all four biological replicates, the average signal intensity of proteins was calculated. Significant log‐fold changes in protein expression values in three different groups (‘NPNT vs EV’, ‘EV_rmNPNT_ vs EV’, and ‘RGE vs RGE‐AIA’) were analyzed further.

### Immunoblotting

The protein concentration of the whole‐cell lysates was measured by Bio‐Rad protein assay (Bio‐Rad, Hercules, CA, USA; Cat: 500‐0006). A total of 50 μg protein was loaded in NuPAGE Novex 10% (Invitrogen, Carlsbad, CA, USA; Cat: NP0301BOX). Protein transferred to a PVDF membrane was further incubated with primary antibodies: p38 MAPK (1 : 1000) (CST, Cat: 9212) and phospho‐p38 MAPK (1 : 1000) (CST, Cat: 9211). Bound primary antibodies were detected using an appropriate HRP‐linked secondary antibody (Dako, Santa Clara, CA, USA; Cat: P0447 or P0399) and imaged using Supersignal West Femto substrate (Pierce, Cat: 34096) with the Odyssey Fc system (Li‐Cor biosciences, Lincoln, NE, USA). Western blots were quantified using image studio 3.1 software (Li‐Cor biosciences). Statistical analyses were performed using two‐tailed Student's *t*‐tests assuming equal variance.

### Ingenuity pathway analysis

Ingenuity pathway analysis (Qiagen, Hilden, Germany) is a web‐based program which uses algorithms to connect protein expression values to its corresponding biological response. The RPPA analysis resulted in a list of differentially expressed proteins (log‐fold change) between the RGE and RGE‐AIA group, which was further analyzed by IPA to identify the cellular function most likely to be affected by the alteration in the NPNT enhancer motif. IPA bases its analysis on already published information about protein networks. A stronger prediction (lower *P*‐value) is made when several proteins are present within the same pathway.

### Cell viability assay

A total of 2500 cells per well were seeded in a 384‐well plate in serum‐free medium for 24 h. Cells were lysed using the Cell Titer‐Glo luminescent cell viability assay kit (Promega, Madison, WI, USA; Cat: G7570). The end point of this assay reports luminescence, which is proportional to ATP generated from cells surviving in serum‐free medium. Cell viability was also measured in 66cl4‐EV cells when incubated with serum‐free medium supplemented with rmNPNT (2 μg·mL^−1^). The p38 MAPK inhibitor BIRB 796 (Axon Medchem, Groningen, Netherlands; Cat: 1358) was used at 4 μm, which was found to be the optimal concentration for 66cl4 and 4T1 cell lines. The p38 MAPK inhibitors LY2228820 (Selleckchem, Munich, Germany; Cat: S1494) and SB203580 (Selleckchem, Cat: S1076) were used at a concentration of 5 μm. The cells were grown in serum‐free medium and simultaneously exposed to the inhibitors for 24 h.

## Results and Discussion

### Cell surface distribution of NPNT in 66cl4 cells

**Figure 1:**
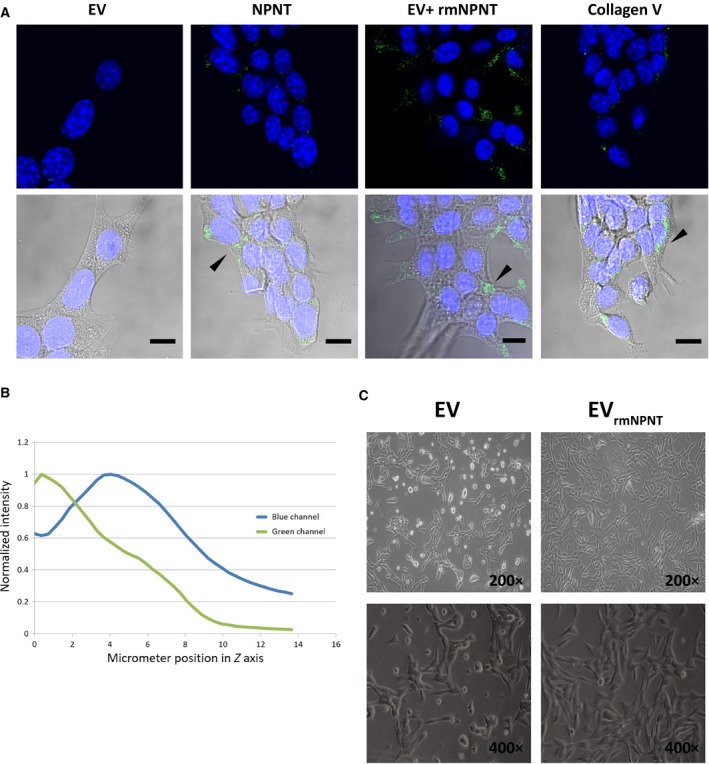
Cell surface distribution of NPNT in 66cl4 cells. (A) Immunofluorescence microscopy showing extracellular NPNT detected on 66cl4 cells expressing wild‐type NPNT and 66cl4‐EV cells when preincubated with rmNPNT for 1 h prior fixing. 66cl4‐EV was used as a negative control. Detection of collagen V on 66cl4‐NPNT cells was used as a positive control. Primary antibodies were visualized with Alexa Fluor 488. Nucleus is stained blue with Hoechst. Scale bar 10 μm. (B) Z profile comparing the green and blue channels was calculated by normalizing mean intensity per slice in the stack for each channel using the image of 66cl4 cells overexpressing NPNT shown above. (C) Brightfield microscopy images of 66cl4‐EV cells growing on uncoated plates (EV) in contrast to rmNPNT‐coated plates (EV_rm_ _NPNT_) at 24 h.

Although NPNT is mostly documented to be an extracellular protein 6, 17, 18, we have recently shown that NPNT is localized intracellularly in the cytoplasm and packed in vesicles/granules in breast cancer tissues and in exosomes isolated from cell lines 14. Our previous findings showing an integrin‐dependent metastasis‐promoting effect also suggest extracellular localization and function of NPNT in breast cancer. To visualize the cell surface distribution of NPNT in 66cl4 cells overexpressing NPNT (66cl4‐NPNT), we imaged the cells using immunofluorescence microscopy (Fig. 1A). In this experimental setup, without permeabilization of the cells, the results showed an extracellular focal distribution of NPNT. 66cl4‐EV cells cultured in serum‐free medium for 24 h were used as a negative control. Recombinant NPNT (2 μg·mL^−1^) added to the 66cl4‐EV cells for 1 h prior to fixing could be detected in a similar location as wild‐type NPNT in 66cl4‐NPNT cells. Collagens are major constituents of ECM, and staining for collagen V showed a similar pattern as staining for NPNT, further demonstrating extracellular localization of NPNT in the 66cl4 cells. Using Z‐stack images of the 66cl4‐NPNT cells, we plotted a Z profile showing the signal intensity in the green (NPNT‐Alexa 488) and blue (nucleus‐Hoechst) channels as a function of distance from the surface of the culture dish (Fig. 1B). The graph shows that the signal derived from NPNT is located below the nucleus, close to the surface of the plate (Video S1). This points to an extracellular localization of NPNT, as has already been shown by others 6, 17, 18. Seeding equal numbers of 66cl4‐EV cells on rmNPNT‐coated plates and uncoated plates showed that presence of rmNPNT increased the proportion of cells that attached and spread out compared to the uncoated plates where a large number of cells still remained rounded at 24 h (Fig. 1C). This is in line with our previous finding showing involvement of NPNT in promoting cell adhesion 14.

### RPPA analysis of NPNT‐mediated signaling

**Figure 2:**
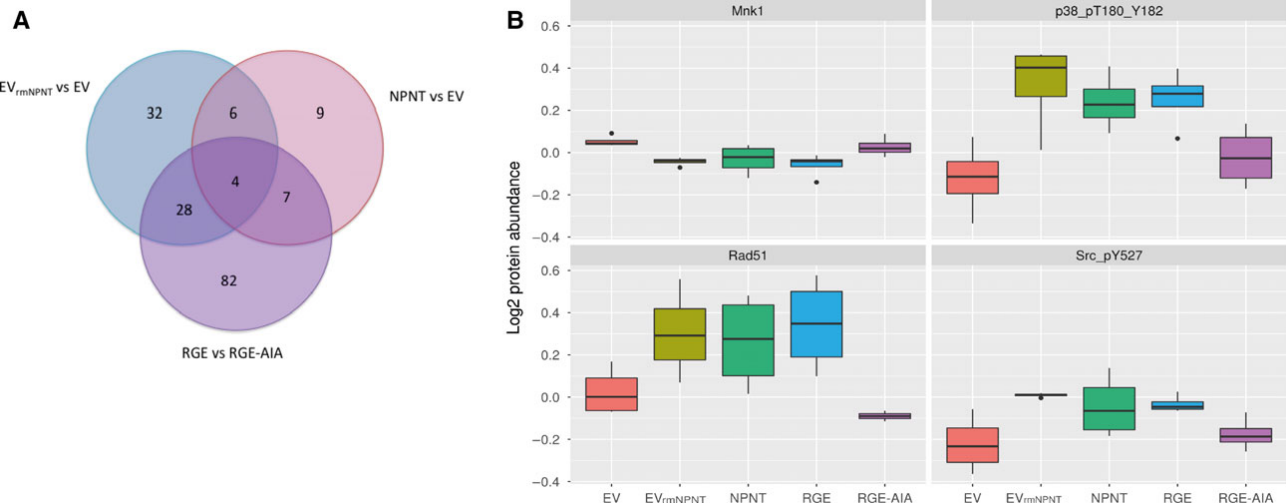
Reverse‐phase protein array analysis of NPNT‐mediated signaling. The Venn diagram includes number of proteins significantly regulated and/or modified (*P* < 0. 05) in all four biological replicates. (A) The pink circle in the Venn diagram, ‘NPNT vs EV’, denotes the log‐fold change values triggered in 66cl4‐NPNT cells in comparison with 66cl4‐EV cells. The blue circle, ‘EV_rm_ _NPNT_ vs EV’, represents 66cl4‐EV cells cultured on rmNPNT (EV_rm_ _NPNT_) in comparison with 66cl4‐EV cells seeded in noncoated wells. The purple circle represents proteins regulated by the integrin‐binding motifs of NPNT; the effect of a single mutation in the RGD motif (RGD → RGE) versus mutations in both RGD and EIE motifs (RGD‐EIE ‐> RGE‐AIA). (B) Box plot showing log2 protein abundance of the four overlapping proteins from the Venn diagram.

Various ECM proteins contribute in establishing the phenotype of mammary epithelial cells and can regulate tissue‐specific function in an autocrine or paracrine manner 19. To elucidate the downstream intracellular signaling effects of extracellular NPNT, we used high‐throughput RPPA functional proteomics that allow the measurement of protein levels and relative amounts of phosphorylated proteins in several samples using 300 different antibodies simultaneously 20, 21. The signal intensity from protein–antibody binding was quantified and used for data analysis. The three circles in the Venn diagram represent (a) the proteins regulated by seeding control cells (66cl4‐EV) on plates coated with rmNPNT (EV_rmNPNT_ vs EV), (b) the proteins regulated by the NPNT overexpression (NPNT vs EV), and (c) the proteins regulated by the integrin‐binding enhancer motif alone (RGE vs RGE‐AIA) (Fig. 2A/Table S1). The four proteins in the overlap between these three comparisons were identified as p38 MAPK, Src, Mnk1, and Rad51 (Fig. 2B) and may represent possible common players of NPNT‐induced signaling. Dual phosphorylation of p38 MAPK at T180 and Y182 activates downstream intracellular signals to regulate growth, differentiation, survival, and respond to stress 22, 23. Src is a downstream effector in integrin signaling, and phosphorylation of Src at Y527 is usually transient and renders the enzyme less active 24, 25. Rad51 is known for its role in DNA repair 26. Mnk1 acts downstream in p38 MAPK signaling pathway 27. The presence of either wild‐type NPNT or NPNT‐RGE increased phosphorylation of p38 MAPK (T180 and Y182) and phosphorylation of Src (Y527), while the double mutant of NPNT did not (Fig. 2B). Rad51 protein levels increased when cells were seeded onto rmNPNT or expressing either wild‐type NPNT or NPNT‐RGE, while cells expressing NPNT with the double mutation did not show any increase in Rad51 protein levels. For Mnk1, the effect was opposite. Compared to 66cl4‐EV control cells, Mnk1 protein levels were reduced in cells seeded onto rmNPNT or expressing either wild‐type NPNT or NPNT carrying the RGD mutation, while cells expressing NPNT mutated in both the RGD and the enhancer motif EIE did not display altered protein levels of Mnk1 (Fig. 2B). Suppression of Mnk1 expression has been reported to increase the eukaryotic transcription initiation factor 4F activity (eIF4F) 28, a factor known to promote survival of breast cancer cells 29. Further studies are required to identify the involvement of transcription factors such as eIF4F, and apoptosis‐regulating proteins influenced by NPNT‐induced signaling. The RPPA analysis suggests that NPNT influences on the total protein levels of Mnk1 and Rad51 and the phosphorylation status of p38 MAPK and Src. These results also point to the importance of the integrin enhancer motif in these regulatory processes.

### NPNT promotes cell viability via its enhancer motif

**Figure 3:**
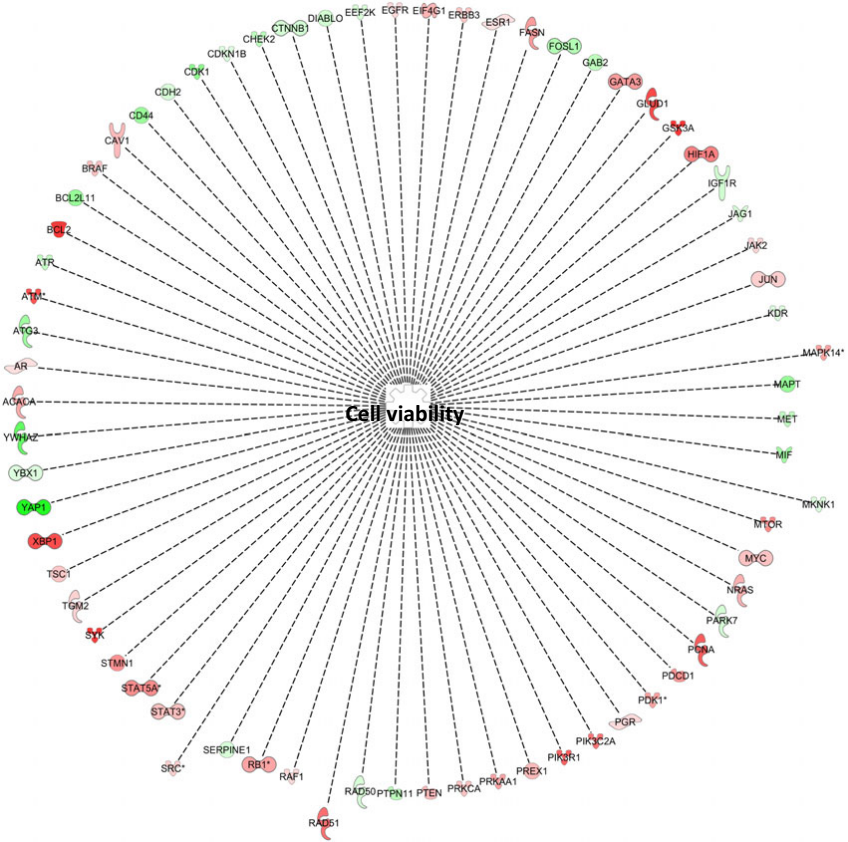
Nephronectin promotes cell viability via its enhancer motif. Representation of the 69 upregulated (red) or downregulated (green) proteins (shown as gene symbols) identified by IPA to have a direct relationship with cell viability. The asterisk indicates that multiple proteins in the dataset file map to a single gene.

Ingenuity pathway analysis (IPA) can recognize the RPPA protein signal intensities and correlate them to their corresponding genes and then predict potential downstream cellular functions. We have utilized the dataset from the ‘RGE vs RGE‐AIA’ group to specifically identify the molecular and cellular functions supported by the integrin‐binding enhancer motif of NPNT. Cell death and survival, cellular growth and proliferation, and cellular development are some of the top categories which were predicted to be influenced by the NPNT enhancer motif (Table 1). In each of the categories, we could investigate further the specific cellular functions using the up‐ and downregulated proteins expression values from the RGE vs RGE‐AIA group. There were 69 proteins pointing toward a role of the NPNT enhancer motif in cell viability (Fig. 3 and Table 1). Rad51, p38 MAPK (shown as MAPK14), Mnk1 (shown as MKNK1), and Src are among those 69 proteins known to influence cell viability (Fig. 3). In line with our results, NPNT has also previously been reported involved in survival of osteoblasts 30. NPNT is known to interact with integrin α8β1 8, 31, and integrins activate survival pathways via PI3K‐kinase or MAPKs 3, 32. Phosphorylated p38 MAPK can have a pleiotropic role, mediating either cell survival or cell death depending on the cell type, disease stage, and type of stimulus 33, 34, 35. Activated p38 MAPK can phosphorylate various transcription factors as well as antiapoptotic (Bcl‐2) and proapoptotic (Bad) proteins 36. In breast cancer, phosphorylated p38 MAPK has been linked to poor outcomes 37. Interestingly, interference with p38 MAPK signaling in cancer cells has been shown to reduce the tumor‐promoting capacities of the microenvironment 38 and to potentiate the effect of conventional chemotherapies (reviewed in 39), and was therefore chosen for further analysis in this study.

**Table 1:** Top predicted molecular and cellular functions. RPPA results from RGE vs RGE‐AIA group were analyzed using the web‐based software application ingenuity pathway analysis (IPA) tool to identify the most significant NPNT‐responsive functions

Categories	Subcategories	*P*‐value	Predicted activation state	No. of molecules
Cell death and survival	Cell viability	2.18E‐44	Increased	69
Cellular growth and proliferation	Colony formation	6.2E‐35	Increased	44
Cellular development	Maturation of cells	1.03E‐24	Increased	32

### NPNT mediates cell viability via p38 signaling pathways

**Figure 4:**
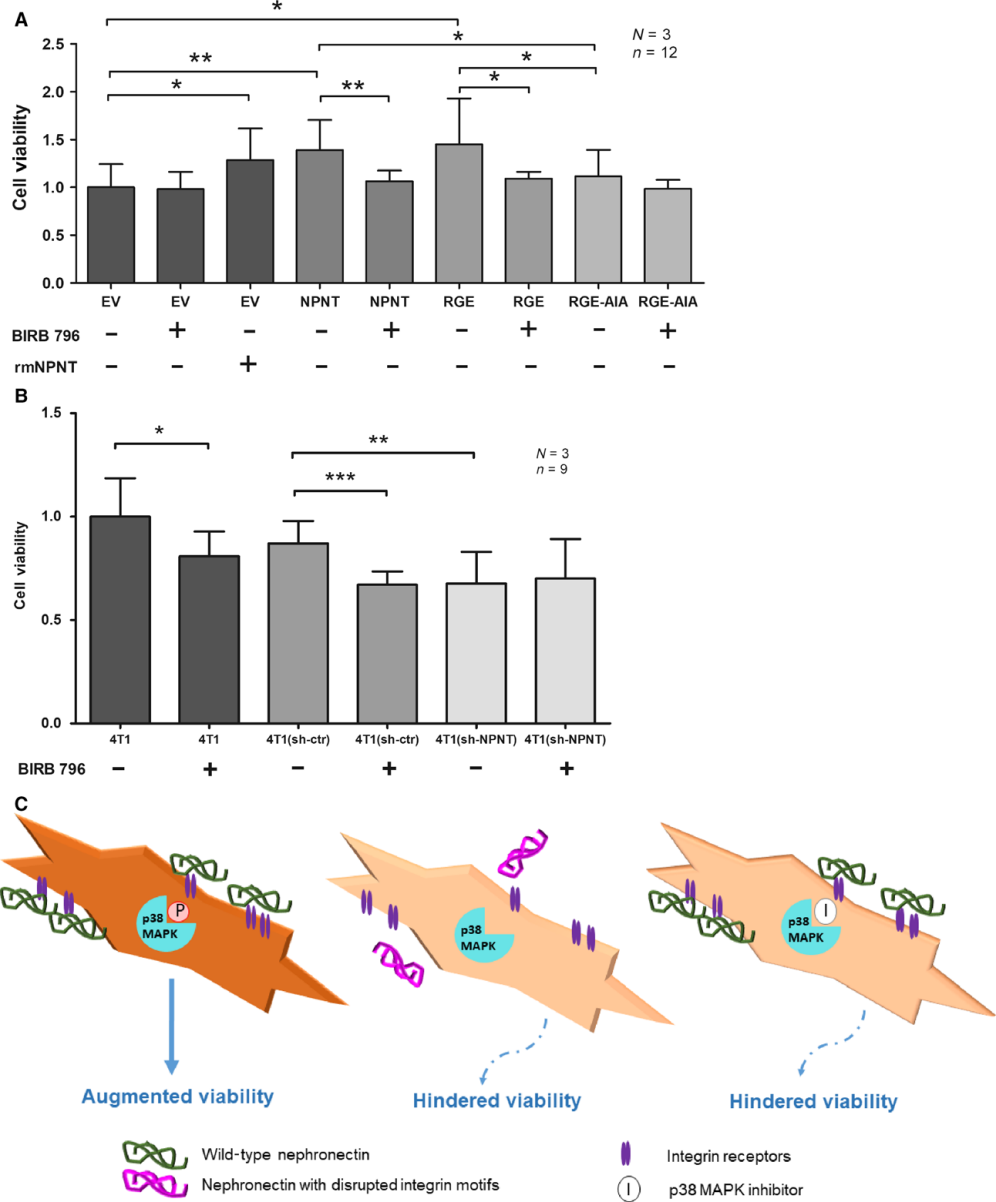
Nephronectin mediates cell viability via p38 signaling pathways. (A) Indicated variants of 66cl4 cells were treated with (±) 4 μm p38 MAPK inhibitor (BIRB 796) for 24 h, in addition to serum deprivation. Where indicated, 66cl4‐EV cells were stimulated by adding 2 μg·mL^−1^ rmNPNT to the cell culture medium. Cell viability was determined using CellTiter‐Glo. (B) Viability of NPNT expressing, 4T1 cells with a NPNT‐targeted short hairpin (sh‐NPNT) and a nontargeting shRNA (sh‐ctr) was tested using CellTiter‐Glo. Significance is tested using a two‐tailed Student's t‐test. *P < 0. 05, **P < 0. 005, ***P < 0. 0001. Error bars represent SD. *N* = number of independent experiments, *n* = total number of replicates in each test group. (C) Illustration summarizing the cellular effects of integrin binding to wild‐type or mutated NPNT via p38 MAPK.

Results from the RPPA and IPA analyses indicated that NPNT and its integrin‐binding motifs could be involved in determining cell viability via p38 MAPK phosphorylation in our study model. An *in vitro* cell viability assay was used to analyze the different 66cl4 cells. An increase in viability was seen when EV cells were incubated with serum‐free medium containing rmNPNT, or when 66cl4 cells were overexpressing wild‐type NPNT. In cells where both integrin‐binding motifs were mutated (RGE‐AIA), there was a reduction in viability compared to cells overexpressing wild‐type NPNT (Fig. 4A/Dataset 1). Exposure of 66cl4‐EV cells to rmNPNT (EV _rmNPNT_) coating for 24 h in serum‐free media stimulated p38 MAPK phosphorylation compared to control cells (Fig. S1a/Dataset 2), thus confirming the results obtained from RPPA. To further explore the involvement of p38 MAPK in NPNT‐induced viability, we utilized the p38 MAPK inhibitor, BIRB 796, known to inhibit all p38 MAPK isoforms *in vitro* 40. The viability of control cells (EV) was unaffected by p38 MAPK inhibition, thereby excluding any off‐target effects that may interfere with viability in this particular assay. On the other hand, a significant decrease in viability was seen in both 66cl4‐NPNT and 66cl4‐NPNT‐RGE cells when phosphorylation of p38 MAPK was inhibited using BIRB 796, while addition of BIRB 796 to cells carrying the double mutation (RGE‐AIA) had no statistically significant effect on cell viability. Similar findings were obtained using the p38 MAPK inhibitors, LY2228820 and SB203580, significantly reducing NPNT‐mediated cell viability (Fig. S2a/Dataset 3). This indicates that p38 MAPK functions downstream of NPNT and regulates viability of the 66cl4 cells. These observations were further validated by comparing the parental 66cl4 cells, with low endogenous NPNT, to the parental 4T1 cells, with high endogenous NPNT levels 12, 14. The 66cl4 cell line generally showed lower p38 MAPK phosphorylation compared to the 4T1 cells (Fig. S1b/Dataset 2). When 4T1 cells were treated with the p38 MAPK inhibitor, viability was markedly reduced (Fig. 4B/Fig. S2). 4T1 cells, expressing a nontargeting shRNA (sh‐ctr), responded similarly as the 4T1 parental cells. Next, we analyzed 4T1 cells with stable shRNA knock‐down of NPNT, 4T1 (sh‐NPNT) 14. The knock‐down generally decreased the cell viability of the 4T1 (sh‐NPNT) cells compared to the parental 4T1 cells. Although treatment with the BIRB 796 did not further decrease the viability (Fig. 4B/Dataset 1), the LY2228820 and SB203580 inhibitors did reduce viability in the 4T1 (sh‐NPNT) cells (Fig. S2b/Dataset 3).

Taken together, these results demonstrate a role for NPNT and its integrin‐binding motifs, in particular the EIE‐enhancer motif, in the induction of p38 MAPK signaling and cell viability. There are four members of the p38 family (p38α, p38β, p38γ, and p38δ), of which p38α (MAPK14) is best studied and expressed in most cell types 41. However, further investigation is needed to elaborate on the role specific role of the different p38 isoforms. Results from the current study are summarized in Fig. 4C and show that NPNT can activate p38 MAPK and by that promote viability in 66cl4 breast cancer cells. The requirement of the NPNT EIE‐enhancer motif in the activation of p38 MAPK is a novel finding, making this a potential drug target in tumors with high NPNT expression. Interestingly, though the RGD motif has shown great promise as a therapeutic target 2, drugs such as cilengitide have failed in clinical trials due to lack of efficiency 42. Based on the current findings, we therefore suggest that dual targeting of the RGD and EIE‐enhancer motif could prove to be more efficient for cancers with high NPNT levels.

## Author contributions

TSS and GS conceived and supervised the study. JT and SNM designed the experiments. JT and KC performed the experiments and analyzed the data. All authors have contributed equally to writing of the manuscript and revisions.

## Conflict of interest

The authors declare no conflict of interest.

## Data Accessibility

Dataset 1: Raw data for Fig. 4; Dataset 2: Raw data for Fig. S1; Dataset 3: Raw data for Fig. S2.

## Supporting information


**Fig. S1.** Phosphorylation of p38 MAPK in the presence of NPNT (a) Immunoblotting for detecting phosphorylation levels of p38 MAPK using whole cell lysates made of 66cl4‐EV and 66cl4‐EV_rmNPNT._ cultured under serum‐free conditions for 24 h. (b) Immunoblotting for phospho‐p38 MAPK and total‐p38 MAPK level using lysates from mother cell lines, 66cl4 and 4T1, grown on uncoated plates for 24 h in serum‐free conditions. Quantification of optical density represents the mean of three independent experiments. Significance is tested using a two tailed Student's *t*‐test assuming equal variance. *P < 0.05, **P < 0.005, ***P < 0.0001. Error bars represent SD.

**Fig. S2.** NPNT mediates cell viability via p38 signaling pathways (a) Indicated variants of 66cl4 cells were treated with (+/−) 5 μm p38 MAPK inhibitor (LY2228820 or SB203580) for 24 h, in addition to serum deprivation. Cell viability was determined using CellTiter‐Glo. (b) Viability of NPNT expressing, 4T1 cells with an NPNT‐targeted short hairpin (sh‐NPNT) and a nontargeting shRNA (sh‐ctr) was tested upon incubating cells with (+/−) 5 μm p38 MAPK inhibitor (LY2228820 or SB203580) for 24 h. Significance is tested using a two tailed Student's *t*‐test. *P < 0.05, **P < 0.005, ***P < 0.0001. Error bars represent SD. N = number of independent experiments, n = total number of replicates in each test group.

**Table S1.** RPPA details. List of differentially expressed proteins shown in the Venn diagram, Fig. 2a.

**Video S1.** *Z*‐stack sections in 66cl4‐NPNT cells were compiled to visualize the signal for NPNT (green channel). Each image in the section has a voxel depth of 0.36 μm. Assuming the reflection coming from the culture plate to be zero, we get the highest intensity for NPNT‐Alexa 488 at the 4th section. This means we get the highest intensity signal for NPNT at 1.4 μm (0.36*4).
