# Supplementary material for: Nephronectin mediates p38 MAPK‐induced cell viability via its integrin‐binding enhancer motif
Source: FEBS Open Bio. 2018 Nov 15;8(12):1992–2001. doi: 10.1002/2211-5463.12544 (PMC6275265; doi:10.1002/2211-5463.12544)
Supplement: Supplementary file 1 — Fig. S1. Phosphorylation of p38 MAPK in the presence of NPNT (a) Immunoblotting for detecting phosphorylation levels of p38 MAPK using whole cell lysates made of 66cl4‐EV and 66cl4‐EVrmNPNT. cultured under serum‐free conditions for 24 h. (b) Immunoblotting for phospho‐p38 MAPK and total‐p38 MAPK level using lysates from mother cell lines, 66cl4 and 4T1, grown on uncoated plates for 24 h in serum‐free conditions. Quantification of optical density represents the mean of three independent experiments. Significance is tested using a two tailed Student's t‐test assuming equal variance. *P < 0.05, **P < 0.005, ***P < 0.0001. Error bars represent SD. [file FEB4-8-1992-s001.pdf]

Figure S1

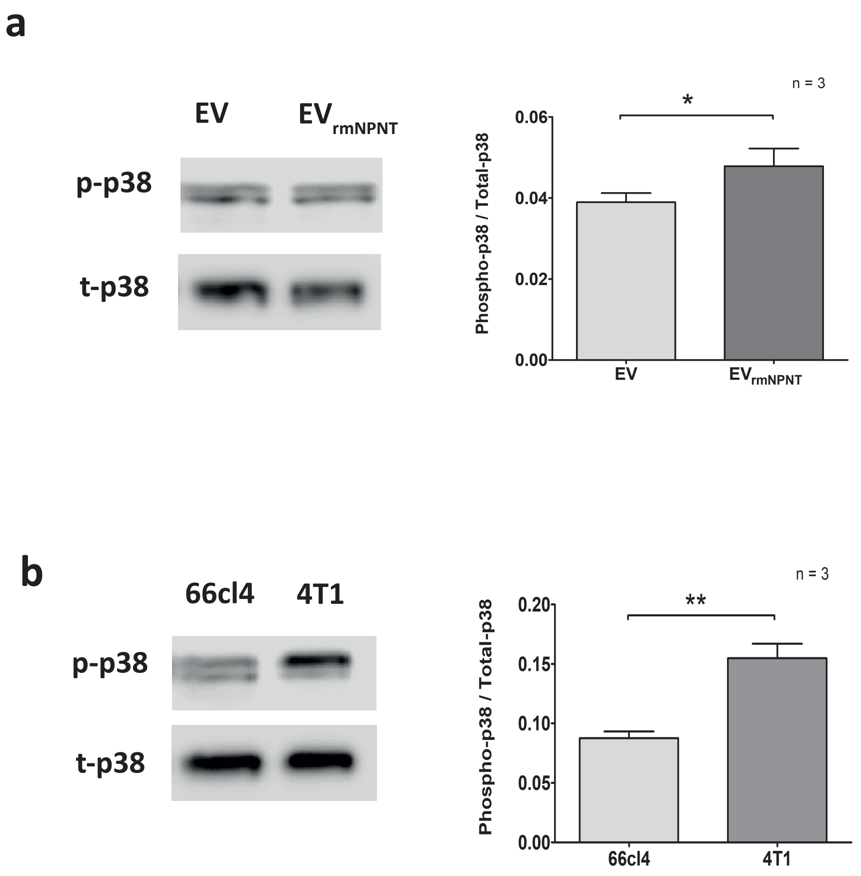

Fig. S1: *Phosphorylation of p38 MAPK in the presence of NPNT* (a) Immunoblotting for detecting phosphorylation levels of p38 MAPK using whole cell lysates made of 66cl4-EV and 66cl4-EV_{rmNPNT}. cultured under serum-free conditions for 24 hours. (b) Immunoblotting for phospho-p38 MAPK and total-p38 MAPK level using lysates from mother cell lines, 66cl4 and 4T1, grown on uncoated plates for 24 hours in serum free conditions. Quantification of optical density represents the mean of three independent experiments. Significance is tested using a two tailed Student's t-test assuming equal variance. *P<0. 05, **P<0. 005, *** P<0. 0001. Error bars represent SD.
