# Supplementary material for: Nephronectin mediates p38 MAPK‐induced cell viability via its integrin‐binding enhancer motif
Source: FEBS Open Bio. 2018 Nov 15;8(12):1992–2001. doi: 10.1002/2211-5463.12544 (PMC6275265; doi:10.1002/2211-5463.12544)
Supplement: Supplementary file 2 — Fig. S2. NPNT mediates cell viability via p38 signaling pathways (a) Indicated variants of 66cl4 cells were treated with (+/−) 5 μm p38 MAPK inhibitor (LY2228820 or SB203580) for 24 h, in addition to serum deprivation. Cell viability was determined using CellTiter‐Glo. (b) Viability of NPNT expressing, 4T1 cells with an NPNT‐targeted short hairpin (sh‐NPNT) and a nontargeting shRNA (sh‐ctr) was tested upon incubating cells with (+/−) 5 μm p38 MAPK inhibitor (LY2228820 or SB203580) for 24 h. Significance is tested using a two tailed Student's t‐test. *P < 0.05, **P < 0.005, ***P < 0.0001. Error bars represent SD. N = number of independent experiments, n = total number of replicates in each test group. [file FEB4-8-1992-s002.pdf]

Figure S2

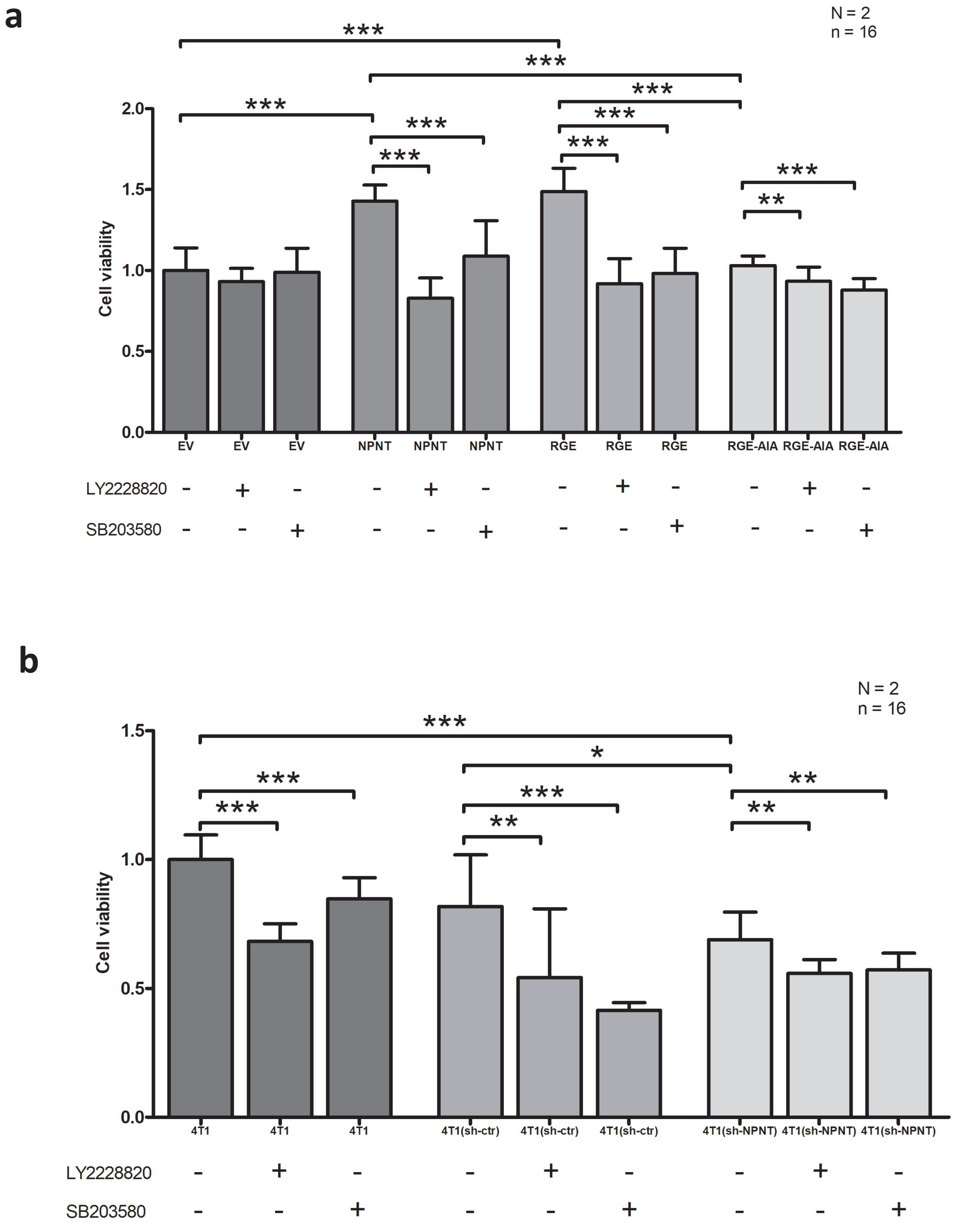

Fig. S2: *NPNT mediates cell viability via p38 signaling pathways* (a) Indicated variants of 66cl4 cells were treated with (+/-) 5 µM p38 MAPK inhibitor (LY2228820 or SB203580) for 24 hours, in addition to serum deprivation. Cell viability was determined using CellTiter-Glo. (b) Viability of NPNT expressing, 4T1 cells with a NPNT-targeted short hairpin (sh-NPNT) and a non-targeting shRNA (sh-ctr) was tested upon incubating cells with (+/-) 5 µM p38 MAPK inhibitor (LY2228820 or SB203580) for 24 hours. Significance is tested using a two tailed Student's t-test. *P<0. 05, **P<0. 005, *** P<0. 0001. Error bars represent SD. N = number of independent experiments, n = total number of replicates in each test group.
