# Supplementary material for: Nephronectin mediates p38 MAPK‐induced cell viability via its integrin‐binding enhancer motif
Source: FEBS Open Bio. 2018 Nov 15;8(12):1992–2001. doi: 10.1002/2211-5463.12544 (PMC6275265; doi:10.1002/2211-5463.12544)
Supplement: Supplementary file 3 — Table S1. RPPA details. List of differentially expressed proteins shown in the Venn diagram, Fig. 2a. [file FEB4-8-1992-s003.pdf]

Details of RPPA

Gene	Antibody	logFC_EVR-EV	P.Value_EVR-EV	logFC_NPNT-EV	P.Value_NPNT-EV	logFC_ΔRGD-ΔRGDΔEIE	P.Value_ΔRGD-ΔRGDΔEIE
OCT.4	Src_pY416	0.010841896	0.859928626	-0.199031547	0.003577582	-0.121005703	0.059319053
NDRG1	NDRG1_pT346	0.12985959	0.128585858	0.204107433	0.021390075	-0.123593956	0.147036728
RPS6K	p90RSK_pT573	0.02305851	0.592315755	0.135998935	0.004232499	0.056035382	0.200561927
AXL	Axl	-0.093680316	0.08622482	-0.119237838	0.032392378	-0.051052068	0.337759623
RPS6KA1	RSK	0.026772041	0.488312547	0.101693825	0.014034972	0.030206985	0.4349922
PEA15	PEA.15	-0.018206461	0.743911619	-0.264138063	9.58E-05	0.035186817	0.5292585
PRKCB	PKC.b.II_pS660	0.076711007	0.346638843	0.179993258	0.034649857	0.023803343	0.768078618
PPIF	Cyclophilin.F	0.102598484	0.127809535	0.207899401	0.004179538	-0.017494291	0.789538231
PARP1	PARP1	-0.021901747	0.687525881	0.149009265	0.011356545	0.002638478	0.961279244
EIFGE	eIF4E	-0.286541849	8.72E-07	-0.076171619	0.082225137	0.006917994	0.869923258
EIF4E	eIF4E_pS209	-0.177262142	0.009353489	-0.048551114	0.442004424	-0.119593387	0.067189766
HISTH3	DM.Histone.H3	-0.16586435	0.003043858	-0.072392125	0.158675516	-0.079457394	0.123613483
VTCN	B7.H4	-0.152418251	0.005817337	-0.041814032	0.409222732	-0.057345952	0.261107587
PAK1	PAK1	-0.118556383	0.01778206	-0.048009811	0.309513559	0.044774286	0.342476515
COG3	COG3	-0.113149353	0.001751114	-0.046011593	0.159854093	-0.030897134	0.338955529
IRF1	IRF.1	-0.1089811	0.008086753	-0.004362232	0.907896987	0.026891125	0.478354829
SDHA	SDHA	-0.107478327	0.003070932	-0.030659898	0.350851124	0.038447366	0.244825362
INPP4B	INPP4b	-0.105221053	0.039265664	-0.064828556	0.189969325	0.043837408	0.370103022
BCL2A1	Bcl2A1	-0.09759928	0.02797047	-0.005585186	0.893810474	0.033001802	0.43360579
TUBAIA	D.a.Tubulin	-0.093536589	0.01949652	0.018707464	0.618200662	-0.04663383	0.221109173
COL6A1	Collagen.VI	-0.084837539	0.04511025	-0.039111657	0.337167211	-0.024092646	0.551626931
FOXM1	FoxM1	-0.083951112	0.026349329	-0.068037041	0.0664093	0.01509842	0.671797895
DPP4	CD26	-0.083485563	0.009079862	-0.000123805	0.996639389	0.024442003	0.409556239
RPS6KB1	p70.S6K_pT389	-0.079206902	0.010391297	-0.013761868	0.629924797	0.025827951	0.36919508
RAB11A	Rab11	-0.070061242	0.031784174	0.001940262	0.949808319	0.059461926	0.064365285
PAICS	PAICS	-0.064316894	0.033623982	-0.00404692	0.887619004	0.009274613	0.746290474
PRKAA2	AMPK.a2_pS345	-0.061634967	0.040693031	-0.029870303	0.302511146	0.045301703	0.123851561
XPA	XPA	0.091776164	0.046278508	0.004005798	0.927221526	0.002448143	0.955479891
PDCD4	Pdcd4	0.111694673	0.006121611	0.042539625	0.25882744	-0.021915465	0.556288155
EGFR	EGFR_pY1173	0.142704638	0.003222898	0.015594573	0.720037385	0.016245895	0.708903931

BRAF	B.Raf	0.143136761	0.022107232	0.079508624	0.184321579	-0.05261506	0.373971176
KIT	c.Kit	0.145101499	0.003407721	-0.021682657	0.627007383	0.03996869	0.373618497
XPF	XPF	0.147511205	0.024840166	0.065463163	0.295634318	0.029642067	0.632231381
CHEK2	Chk2_pT68	0.153235863	0.002935154	-0.000662144	0.988538896	-0.027069286	0.558670316
BAK1	Bak	0.163774926	0.000961101	-0.019257299	0.656570781	-0.042127032	0.335009612
RBM15	RBM15	0.163888082	0.043168199	0.077201593	0.322207772	0.14938796	0.063180639
ACACA	ACC1	0.186115032	0.001691448	0.094212787	0.082826259	0.086304025	0.110061567
ADAR1	ADAR1	0.189157306	0.004716095	0.084933023	0.170540121	-0.060041131	0.327182583
BAD	Bad_pS112	0.19077437	0.015144991	0.06493262	0.37819343	0.005663996	0.938151317
PTGS3	Cox.IV	0.26125845	0.04423033	0.121260188	0.331834374	0.084071436	0.498535376
ATRX	ATRX	0.441560179	0.000288392	0.209134569	0.052482465	0.092194559	0.375148045
ETS1	Ets.1	-0.24623588	0.06927583	-0.05257617	0.68683031	-0.279814761	0.041150018
STAT5A	Stat5a	-0.142443749	0.263266187	-0.046969271	0.708461848	0.282508638	0.033172859
CD44	CD44	-0.136896287	0.058748411	-0.097608962	0.168815912	-0.25418844	0.001293425
PAX8	PAX8	-0.12902807	0.052715487	-0.103487803	0.114480333	-0.155227829	0.022159842
MIF	MIF	-0.123585016	0.055686488	-0.042396682	0.494707367	-0.230868007	0.001087222
RIP	RIP	-0.110898943	0.078537106	-0.065946122	0.283905939	-0.14317919	0.026435731
STMN1	Stathmin.1	-0.106873349	0.090907339	0.062580773	0.311252205	0.325501624	2.36E-05
RPS6	S6	-0.100320062	0.272249847	0.122206427	0.184090583	0.618558608	7.25E-07
JAK2	Jak2	-0.093043419	0.137763077	0.005190019	0.932225448	0.130330179	0.042366766
ERBB3	HER3_pY1289	-0.082071698	0.076120017	0.005554968	0.900713455	0.1704616	0.000875902
RB1	Rb_pS807_S811	-0.080211575	0.198773875	0.084367222	0.177389499	0.272440739	0.000193432
CD274	PD.L1	-0.079250708	0.210127632	-0.040090933	0.520215824	-0.13725498	0.036113823
IRS1	Caspase.8	-0.075656862	0.087739806	-0.042266129	0.32846331	-0.255188473	5.40E-06
ATR	ATR_pS428	-0.074118177	0.123213215	0.014939205	0.749366097	-0.15049661	0.0037357
SLC16A4	MCT4	-0.074049974	0.169962279	-0.033335646	0.529247715	-0.215067668	0.000479245
CHEK2	Chk2	-0.072409715	0.137021591	-0.057555577	0.232693581	-0.166564001	0.001866563
ITGA2	CD49b	-0.070477613	0.293657782	-0.040676332	0.540864426	-0.192002743	0.007923994
LRP6	LRP6_pS1490	-0.06717084	0.1774789	-0.07150005	0.152314813	0.133425125	0.011431101
MAPT	Tau	-0.062395444	0.221092273	-0.076753433	0.135763287	-0.257570846	3.69E-05
ERRFI1	MIG6	-0.062153995	0.366262602	0.016436188	0.809445804	-0.233903709	0.002261128
RPS6	S6_pS240_S244	-0.060497054	0.493076291	-0.132538491	0.141355629	0.583551272	1.17E-06
STAT3	Stat3	-0.055701468	0.371308701	0.046839233	0.450905709	0.190003977	0.00522358

ERCC5	ERCC5	-0.052678636	0.434744702	0.005678998	0.932399452	0.139346193	0.04736543
CDK1	CDK1	-0.051803038	0.516303227	-0.043441031	0.585679241	-0.288282797	0.001413071
PRKCA	PKC.a_pS657	-0.04906814	0.267230377	0.029228294	0.504612536	0.181332531	0.000391586
ATM	ATM	-0.047968961	0.162880714	-0.006457284	0.847514389	-0.077417244	0.029609533
JAG1	Jagged1	-0.047881169	0.134252578	-0.003182651	0.918517383	-0.09818323	0.004363219
EGFR	EGFR	-0.046248481	0.165405283	-0.023547714	0.472412576	0.114797481	0.001819312
AR	AR	-0.046088659	0.062539906	0.018635487	0.435320463	0.066564727	0.009789579
STAT3	Stat3_pY705	-0.035477589	0.360028948	-0.028347099	0.462936009	-0.080594107	0.045551124
CTNNB1	b.Catenin_pT41_S45	-0.033505217	0.312614534	-0.05735598	0.091057145	-0.106275338	0.003559074
PDK1	PDK1	-0.027585885	0.47377725	-0.011244258	0.769131392	0.129243811	0.002589121
AIM1	Aurora.B	-0.026175699	0.577663942	-0.014625343	0.755107967	-0.115335763	0.021136161
RAD50	Rad50	-0.025952339	0.52565683	-0.020336173	0.618310478	-0.094663674	0.02837916
MET	c.Met	-0.024489838	0.544944591	0.000158597	0.996858097	-0.159174455	0.000650794
CCND3	Cyclin.D3	-0.022619542	0.623427285	-0.014910144	0.745795844	-0.211129655	0.000137094
KDR	VEGFR.2	-0.01874291	0.623462434	-0.047708124	0.218562681	-0.08109134	0.042849941
ZAP70	ZAP.70	-0.014832379	0.607562841	-0.016651765	0.564566627	-0.137402663	8.97E-05
HSBP1	HSP27	-0.014718348	0.695397134	-0.031443265	0.405971563	-0.109546266	0.007567776
SRSF1	SF2	-0.010048075	0.839989411	-0.091152176	0.077772327	-0.10414897	0.046192953
SERPINE1	PAI.1	-0.001098447	0.976441751	-0.019783529	0.596081521	-0.096219962	0.01608023
PGR	PR	0.013953375	0.662744049	0.000534999	0.986627586	0.108753278	0.002411584
MYO2A	Myosin.IIa_pS1943	0.016925246	0.895313858	-0.186257632	0.15755114	0.555362518	0.000268208
SRC	Src	0.017168638	0.625888536	-0.031389495	0.375929945	-0.077330952	0.036898784
BCL2	Bcl2	0.021526902	0.814637213	0.1118771	0.23088983	0.608702749	1.21E-06
BABAM1	MERIT40_pS29	0.036231852	0.509208042	0.041995301	0.44504925	-0.149575781	0.011418788
ESR1	ER.a_pS118	0.036511998	0.326137609	-0.006988723	0.849240356	0.076483908	0.047400279
DUSP4	DUSP4	0.037748815	0.384226478	0.05167028	0.23730774	-0.138600055	0.003717624
TSC1	TSC1	0.038402189	0.513236891	0.002359229	0.967794137	0.169550236	0.007886624
MAPK14	p38.MAPK	0.038702778	0.519325362	-0.010594048	0.859341671	0.160476538	0.012899053
CLDN7	Claudin.7	0.03919715	0.33003421	-0.029596217	0.459853325	-0.091553367	0.02991888
ATG3	Atg3	0.039864544	0.326379143	-0.055223704	0.178465566	-0.237954801	5.94E-06
INSRB	IR.b	0.043895959	0.240157216	-0.013944201	0.704832151	-0.242655112	1.30E-06
RPS6	S6_pS235_S236	0.044007241	0.580626135	-0.144956589	0.078674408	0.576292853	3.13E-07
PTEN	PTEN	0.046721648	0.537115398	0.049179606	0.516126559	0.204596137	0.012061449

PIK3R1	PI3K.p85	0.04799038	0.558182895	-0.015673205	0.847784646	0.471462799	8.40E-06
CDKN1B	p27.Kip.1	0.050285245	0.097826752	-0.032333911	0.277828096	-0.064462784	0.037473856
GYS1	Gys_pS641	0.056593654	0.141985965	0.014711703	0.695639825	0.120286154	0.003895393
PIK3C2A	PI3K.p110.a	0.060398374	0.185781613	0.027744048	0.536528747	0.414514452	5.79E-09
RAF1	C.Raf_pS338	0.060651417	0.24215569	-0.030148365	0.556065984	0.116150924	0.03148099
CMC2	Cox2	0.062736902	0.247852474	0.045618427	0.397187055	0.173175932	0.003564618
YWHAZ	X14.3.3.zeta	0.06886934	0.053877355	0.060229985	0.088528565	-0.391894011	1.32E-10
TGM2	Transglutaminase	0.071565479	0.11478092	0.067789233	0.134047521	0.131034142	0.006628571
PREX1	PREX1	0.075007558	0.146032106	0.020748224	0.68048169	0.207242103	0.000431732
CAV1	Caveolin.1	0.077197667	0.191843147	0.073823106	0.211208356	0.203233857	0.00189406
BRAF	B.Raf_pS445	0.079218225	0.120565204	0.016779782	0.735199394	0.184977801	0.001102708
PDK1	PDK1_pS241	0.079914047	0.187143368	0.04371726	0.463967924	0.284910973	8.32E-05
AKT1S1	PRAS40	0.089180841	0.15782481	0.089896102	0.154673593	-0.147451553	0.024587132
HIF1A	Hif.1.alpha	0.090887126	0.453496951	0.059749817	0.620812794	0.380133714	0.004359705
PECAM1	CD31	0.095586677	0.259966388	0.086221984	0.308209879	0.25213528	0.006030841
MTOR	mTOR	0.096963435	0.256533156	0.071651694	0.398472072	0.354568269	0.000344771
IGF1R	IGF1R_pY1135_Y1136	0.104384733	0.067000044	-0.066714108	0.230615232	-0.116067304	0.04354526
FASN	FASN	0.129915823	0.215420347	0.116645715	0.264331341	0.299338935	0.007766726
NRAS	N.Ras	0.162977207	0.090284241	0.075807072	0.418127123	0.232094005	0.019527667
PTPN11	SHP.2_pY542	0.164667579	0.052655471	-0.075269965	0.358503838	-0.167158762	0.049474038
H2BFM	Ubq.Histone.H2B	0.175192546	0.077538719	0.070173058	0.465456957	0.225431746	0.026399824
UGT1A	UGT1A	0.191734199	0.133091477	0.117269608	0.350081848	0.282680731	0.031551219
GCLM	GCLM	0.229237306	0.052816698	0.221633358	0.060456045	0.288035863	0.01751469
TUFM	TUFM	0.252053172	0.057169679	0.201571544	0.122453915	0.33866827	0.013290559
BIRC3	c.IAP2	0.254490259	0.06560264	0.166646811	0.217269485	0.367451273	0.010616151
XBP1	XBP.1	0.272913156	0.069291241	0.208365731	0.158644233	0.535795307	0.001156009
GLUD	Glutamate.D1.2	0.299109662	0.053810036	0.257603361	0.093062613	0.547029623	0.0012187
RAD51	Rad51	0.277689076	0.02080928	0.236844685	0.045004947	0.432818593	0.000833905
MKNK1	Mnk1	-0.095639723	0.012035793	-0.085073612	0.023390492	-0.085685561	0.022524168
SRC	Src_pY527	0.231436637	0.001097655	0.17804457	0.008408807	0.142631082	0.029880155
MAPK14	p38_pT180_Y182	0.443183228	0.002851851	0.361230351	0.011944446	0.277404051	0.046704223
ENY2	ENY2	0.149053983	0.00124477	-0.02771387	0.495878983	-0.474792149	8.88E-11
BCL2L11	Bim	0.124716628	0.011516864	-0.085089951	0.072811718	-0.258145766	1.09E-05

CD29	CD29	0.15358763	0.005325362	-0.017401399	0.728250087	-0.283137408	1.10E-05
MYC	c.Myc	-0.129011755	0.010135304	0.055706739	0.23598644	0.179104929	0.000782038
ATM	ATM_pS1981	0.381164231	0.010504675	0.217610246	0.123657941	0.522576053	0.00092565
EIF4G1	eIF4G	-0.212356374	0.001424972	0.062187281	0.294628816	0.221352744	0.000984505
ULK1	ULK1_pS757	-0.199175311	0.002312556	0.026743305	0.646480492	-0.205217527	0.001804423
GSK3A.B	GSK.3a.b	0.372883033	0.041719476	0.286413764	0.110572232	0.607912199	0.001959158
ROCK1	Rock.1	-0.242367023	0.009100975	-0.103112645	0.235109657	-0.293481519	0.00224024
STAT5	Stat5_pY694	0.215838245	0.040461951	0.093388214	0.355454213	0.337919044	0.002580404
ACACA	ACC_pS79	0.205118203	0.018568009	0.127179405	0.128537583	0.248922301	0.005462974
RB1	Rb	-0.130175437	0.007633018	-0.003772481	0.932662909	-0.134948774	0.005960365
KAT2A	GCN5L2	-0.08829456	0.025170812	0.012357989	0.739159042	0.109826962	0.006844985
SYK	Syk	0.541974466	0.011505754	0.280328764	0.166820626	0.585274823	0.00697943
DIABLO	Smac	0.082509975	0.045028366	0.019199089	0.625068235	-0.115445494	0.007104392
JUN	c.Jun_pS73	0.158248062	0.003726413	-0.03844123	0.436988918	0.143889973	0.007372482
MSI2	MSI2	-0.17692707	0.020174509	-0.064035668	0.373263454	-0.199146333	0.01004647
FOSL1	FRA.1	-0.154287126	0.044492168	-0.019863505	0.785872956	-0.191544175	0.014863628
PCNA	PCNA	0.620859146	0.004079343	0.32659972	0.104759754	0.489294325	0.019050376
PDCD1	Pdcd.1L1	0.335537983	0.017022898	0.136346787	0.304237999	0.318604512	0.02260446
NOTCH3	Notch3	0.166052152	0.026493552	-0.054885842	0.439011328	0.169032997	0.024181782
PRKAA1	AMPKa_pT172	0.285954274	0.021916238	0.084663349	0.471738191	0.275306448	0.02668986
VASP	VASP	-0.156321797	0.000400558	-0.048975058	0.202158399	-0.087625105	0.028289666
YBX1	YB1_pS102	0.102756836	0.005042063	0.007615141	0.818721386	-0.076032648	0.030677745
PIK3BC	PI3K.p110.b	0.20484715	0.048820631	0.132525047	0.190418389	0.225736442	0.031487495
CDC25C	cdc25C	-0.11974777	0.019577263	-0.026905188	0.576129218	-0.107985443	0.033212529
PARK7	DJ1	0.115102352	0.019151894	-0.042893082	0.355075001	-0.10269459	0.034278739
ANXA7	Annexin.VII	0.157211084	0.000235721	0.007594313	0.832827213	-0.075894132	0.04463264
YAP1	YAP_pS127	0.153775205	0.06260462	0.338260896	0.000298365	-0.562937031	4.29E-07
GAB2	Gab2	0.031425789	0.19948335	0.132269322	1.56E-05	-0.162008107	9.43E-07
WIPI1	WIPI1	-0.003775698	0.9272142	-0.212272458	3.77E-05	0.205680987	5.50E-05
GATA3	GATA3	0.136128316	0.178527637	0.209234922	0.044310129	0.298104944	0.006111846
CDH2	N.Cadherin	-0.015071168	0.643795266	-0.09467736	0.007695256	-0.096014657	0.006998801
EEF2K	eEF2K	-0.043976851	0.226344369	-0.075114505	0.045263195	-0.101190184	0.009209645
NF2	Merlin	-0.057018217	0.068881569	0.070416159	0.027557477	-0.064941559	0.040454608

GSK3A.B	GSK.3a.b_pS21_S9	0.221664941	0.001317337	0.130855373	0.040258951	0.099491426	0.111313462
BECN1	Beclin	0.333324662	0.00691202	0.310526734	0.01098597	0.152806931	0.184333348
NDUFB4	NDUFB4	0.248244085	0.002260311	0.19237917	0.013612735	0.051945731	0.475115216
GLS	Glutaminase	-0.136492991	0.000865031	-0.096104984	0.012488969	-0.008545856	0.810407438
EIF4EBP1	X4E.BP1	0.2011071	0.000608838	0.112881084	0.034534588	0.004296776	0.932244551
LC3AB	LC3A.B	0.306583529	0.000415637	0.216171181	0.007607768	-0.001510107	0.983740673
